# Application of Machine Learning Techniques for the Diagnosis of Obstructive Sleep Apnea/Hypopnea Syndrome

**DOI:** 10.3390/life14050587

**Published:** 2024-05-02

**Authors:** Oscar Bedoya, Santiago Rodríguez, Jenny Patricia Muñoz, Jared Agudelo

**Affiliations:** 1 School of Systems Engineering and Computer Science, Universidad del Valle, Cali 760032, Colombia; rodriguez.santiago@correounivalle.edu.co; 2 Hospital Universitario del Valle, Cali 760032, Colombia; drajennylombo@gmail.com; 3 School of Internal Medicine, Universidad Libre—Seccional Cali, Cali 760032, Colombia; jared.agudelo1@gmail.com

**Keywords:** artificial intelligence, ensemble methods, machine learning, neural networks, obstructive sleep apnea

## Abstract

Obstructive sleep apnea/hypopnea syndrome (OSAHS) is a condition linked to severe cardiovascular and neuropsychological consequences, characterized by recurrent episodes of partial or complete upper airway obstruction during sleep, leading to compromised ventilation, hypoxemia, and micro-arousals. Polysomnography (PSG) serves as the gold standard for confirming OSAHS, yet its extended duration, high cost, and limited availability pose significant challenges. In this paper, we employ a range of machine learning techniques, including Neural Networks, Decision Trees, Random Forests, and Extra Trees, for OSAHS diagnosis. This approach aims to achieve a diagnostic process that is not only more accessible but also more efficient. The dataset utilized in this study consists of records from 601 adults assessed between 2014 and 2016 at a specialized sleep medical center in Colombia. This research underscores the efficacy of ensemble methods, specifically Random Forests and Extra Trees, achieving an area under the Receiver Operating Characteristic (ROC) curve of 89.2% and 89.6%, respectively. Additionally, a web application has been devised, integrating the optimal model, empowering qualified medical practitioners to make informed decisions through patient registration, an input of 18 variables, and the utilization of the Random Forests model for OSAHS screening.

## 1. Introduction

Obstructive sleep apnea/hypopnea syndrome (OSAHS) is the most common respiratory disorder in humans and is characterized by the complete absence (apnea) or partial absence (hypopnea) of respiratory flow during sleep. An estimated one billion adults aged 30–69 years worldwide have OSAHS, while the number of people with moderate and severe OSAHS may be as high as 425 million [1]. This disease has a significant economic impact on health systems and society. The healthcare cost associated with OSAHS includes the direct costs of diagnosis and treatment, as well as the indirect costs of associated conditions such as obesity and diabetes, and sequelae such as cardiovascular disease and depression [2]. Patients with sleep disorders are workers who usually present a decrease in productivity at work due to fatigue. OSAHS is now recognized as a worldwide public health problem [3,4].

The diagnosis of OSAHS traditionally relies on polysomnography (PSG), a comprehensive test providing insights into both the severity of OSAHS and sleep architecture, as highlighted in [5]. PSG is administered based on clinical suspicion arising from symptoms like drowsiness, cardiovascular events, and phenotypes indicative of snoring. Diagnosis is confirmed when a patient exhibits an apnea/hypopnea index (AHI) equal to or greater than five, with further classification into mild (5 to 15 events per hour), moderate (16 to 30 events per hour), and severe (more than 30 events per hour) categories based on AHI values. Despite its diagnostic accuracy, PSG poses challenges due to its requirement for overnight evaluation in a sleep laboratory, specialized instruments, and trained personnel. These factors contribute to the relative expense, labor intensity, and technical complexity of PSG, limiting its accessibility to a broad patient population in the face of high demand [6]. To address these limitations, simplified methods focusing solely on respiratory variables, such as cardiorespiratory monitoring or pulse oximetry, have been developed. However, these alternatives, while more accessible, do not evaluate sleep quality, tend to underestimate respiratory disorders, and lack the capability to assess non-respiratory sleep conditions comprehensively. Moreover, in cases of high clinical suspicion of OSAHS where alternative methods yield negative or technically deficient results, PSG remains the gold standard. Thus, the imperative emerges to explore avenues for diagnosing OSAHS without exclusive reliance on polysomnography, enabling accurate syndrome diagnosis based on patient data.

The challenge of diagnosing OSAHS has been addressed through various artificial intelligence techniques that circumvent the reliance on polysomnography. In [7], a dataset comprising 313 patients from the Dream Centers of the universities of Foggia and Milan was utilized. Each patient was characterized by 19 variables, selected from a pool of 32 attributes obtained through diverse strategies for feature selection. The variables encompassed demographic factors such as age, sex, body mass index, Mallampati score, as well as Boolean variables indicating the presence of comorbidities such as asthma, hypertension, diabetes, and dyslipidemia, among others. The applied techniques included support vector machines and Random Forests, both aimed at predicting the severity of OSAHS. According to the findings, the model employing the support vector machine technique achieved an area under the Receiver Operating Characteristic (ROC) curve of 65.0% and an accuracy of 44.7%. Similarly, the model employing Random Forests achieved an area under the ROC curve of 63.7% with an accuracy of 44.1%.

Another work addressing OSAHS diagnosis is presented in [8]. In this case, a comprehensive study involving 3343 patients at Taipei Medical University Hospital in Taiwan was conducted. The research employed the fuzzy decision tree technique, and to address imbalances in the data distribution within the training and test sets, the SMOTE (Synthetic Minority Over-sampling Technique) over-sampling strategy [9] was implemented. Each individual in the study was characterized by 18 attributes, encompassing anthropometric measures such as gender, age, weight, height, systolic and diastolic pressure, as well as head and neck circumference. Additionally, variables derived from questionnaires assessing anxiety indices, depression indices, and daytime sleepiness scales were incorporated into the analysis. The reported outcomes underscore the efficacy of the fuzzy decision tree model, achieving an accuracy of 48.2% in predicting the severity of OSAHS. Notably, when employing the SMOTE technique for data balancing, the accuracy significantly improved to 81.8%.

In the realm of techniques used for OSAHS diagnosis, neural networks also play a significant role. In [10], a multilayer perceptron neural network was employed, trained using the Bayesian regularization method. Each patient was uniquely represented by four attributes: gender, age, body mass index, and snoring status. Through experimentation, the optimal network topology was determined to feature a 4-20-1 configuration, consisting of four neurons in the input layer, a hidden layer with 20 neurons, and a single neuron in the output layer. The neural network achieved a diagnostic accuracy of 86.6%. This study utilized data from 201 individuals for both training and testing, including 140 cases with a positive OSAHS diagnosis and 61 individuals without the condition. The dataset was sourced from individuals undergoing evaluation at the sleep clinic of Mevlana University in Turkey, all of whom presented with suspected OSAHS.

Some of the research related to OSAHS diagnosis is distinguished by their utilization of datasets with a limited number of patients. For instance, the study referenced in [11] utilized data from 86 patients admitted to the sleep laboratory at Hospital Vila Nova de Gaia in Portugal. Among these patients, 45 received a diagnosis of OSAHS, while 41 were deemed healthy. Among the 45 OSAHS-diagnosed patients, 17 presented mild OSAHS, 15 had moderate OSAHS, and 13 had severe OSAHS. The primary focus of this research was on training models to ascertain the positive or negative diagnosis of OSAHS, without predicting the degree of severity. Attributes representing each patient were selected from the 33 variables employed in [12], encompassing demographic information, medical history, physical examinations, and comorbidity details. A subset of six variables was chosen for model training, including obesity, neck circumference, abdominal circumference, gender, witnessed apneas, and alcohol consumption before sleep. The research employed Naive Bayes (NB) and tree-augmented Naive Bayes (TAN) techniques. As per the reported results, the Naive Bayes classifier demonstrated an accuracy of 67.68%, while the tree-augmented Naive Bayes classifier achieved an accuracy of 64.53%.

Traditional statistical methods such as linear regression have also been employed in the context of OSAHS diagnosis. For example, in [13], linear regression was utilized for the positive or negative diagnosis of OSAHS, while multinomial logistic regression was employed for classification based on the severity of OSAHS. This study, conducted within the private healthcare system in Brazil, utilized data from 323 patients, all presenting with sleep disorders such as snoring, insomnia, and excessive daytime sleepiness. Among the participants, 59% were male and 41% were female, with ages ranging from 18 to 79 years. Attributes used to characterize each patient included sociodemographic, clinical, and lifestyle information, with the Epworth scale, measuring daytime sleepiness, which was also implemented. Notably, the models revealed statistically significant associations between variables such as age, body mass index, neck circumference, and witnessed apneas with the severity degree of the disease. Recent studies have favored deep learning algorithms over traditional machine learning methods for detecting OSAHS [14,15,16,17]. For example, in [15], Recurrent Neural Networks, Long Short-Term Memory, and Gated Recurrent Unit were employed, achieving accuracy values of 89.5%, 90%, and 90.29%, respectively.

Many of these studies have constructed their models using clinical variables. However, some of these variables are subjective in nature, including sweating frequency, reported concentration and memory difficulties [7], questionnaire responses [8,10], changes in refreshing sleep, humor alterations, and decreased libido [11]. These subjective measures introduce significant intervariability and low reproducibility, thus reducing the generalizability of the models. Other variables, such as Forced Expiratory Volume 1 (FEV1) measured by spirometry and blood gas pressures [7], are difficult to obtain, further limiting the feasibility of integrating the model into medical practice. 

This article presents OSAHS prediction models utilizing four machine learning techniques on a Colombian patient dataset, with data balancing facilitated by the SMOTE technique. The models are constructed using 18 clinical variables and operate independently of polysomnography. The selection of these variables prioritizes reproducibility by avoiding subjective factors and emphasizes simplicity to facilitate implementation in medical decision-making processes. While various machine learning techniques have been explored for OSAHS diagnosis, a significant gap exists in the Colombian context, where no comparable study has been conducted utilizing data representative of the country’s population. Given the distinct characteristics inherent to each population, employing a dataset reflective of Colombian patients becomes imperative, capturing the specific nuances observed in cases within the country. Thus, this study is aimed at determining the accuracy of machine learning methods when applied to OSAHS diagnosis in the Colombian context. Furthermore, the literature review highlights a deficiency in software facilitating the application of proposed machine learning models by qualified medical personnel. This scarcity impedes the broader utilization of artificial intelligence models for informed decision-making in the medical field. Therefore, another key objective of this research is to investigate whether a web application integrating machine learning models can be developed to empower qualified medical personnel, facilitating the integration of AI into decision-making processes. Importantly, this approach has the potential to prioritize patients who stand to benefit the most from the screening process, consequently reducing the rate of PSG negativity for OSAHS and enhancing both accessibility and cost-effectiveness within the healthcare system.

## 2. Materials and Methods

**Figure 1:**
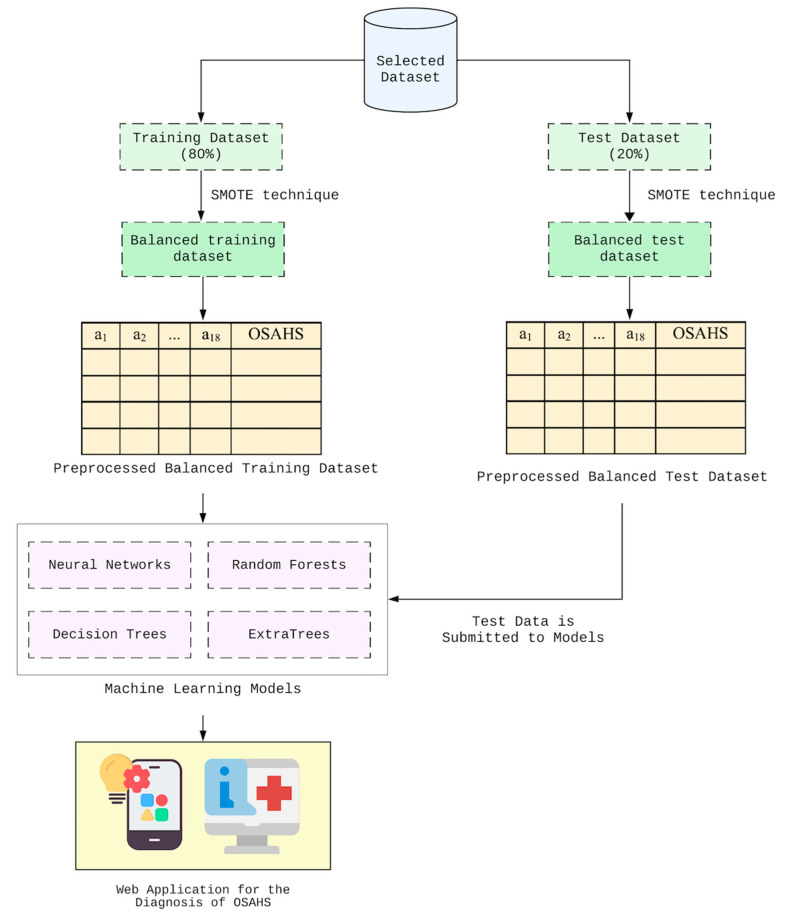
Methodological framework for the diagnosis of patients with OSAHS.

We tested four ML techniques (Neural Networks [18], Decision Trees [19], Random Forests [20], and Extra Trees [21]) based on 18 clinical variables (Table 1), regarding their capacity to predict the result in PSG with respect to OSAHS diagnosis, involving a binary classification (‘OSAHS positive = Hypopnea Index > 5’ and ‘OSAHS negative = Hypopnea Index < 5’). In this research, we chose two machine learning techniques—neural networks and decision trees—based on prior studies, as these methods have demonstrated moderate effectiveness in diagnosing OSAHS. Additionally, we selected two ensemble methods—random forests and Extra Trees. Ensemble methods aggregate the outputs of multiple models to reach a final decision. For example, in the random forest technique, N decision trees are generated, and their outputs are amalgamated using the majority criterion to derive the final classification. Figure 1 illustrates the overarching methodology employed in this study to derive machine learning models. As depicted, the chosen dataset undergoes an 80%/20% split for model training and testing, respectively. Due to the imbalance between positive and negative diagnoses in the dataset, we applied the SMOTE technique, which involves oversampling by generating synthetic instances for the minority class. Importantly, oversampling is performed after segregating the original dataset into training and test sets, ensuring that synthetic instances do not incorporate data from the test set. Each patient is characterized by 19 attributes, encompassing 18 independent variables such as age, gender, body mass index, and comorbidities like diabetes, asthma, and rhinitis, among others. Additionally, a dependent variable assumes values corresponding to OSAHS positive or OSAHS negative. Finally, we developed a web application showcasing the model with superior discrimination and screening capabilities, as assessed by the Area Under the Receiver Operating Characteristic curve (AUROC) and sensitivity, respectively, so it can be applied in medical practice for OSAHS screening.

### 2.1. Dataset and Ethics Declarations

The dataset used comprises records of 601 adults (age > 18 years) evaluated between 2014 and 2016 at a specialized sleep medical center in Cali, Colombia. Clinical suspicion prompted the admission of these patients for confirmation through diagnostic PSG, conducted with the Philips Respironics Alice-6 Diagnostic Sleep System. Notably, the dataset only included individuals with a minimum of four hours of sleep (TST > 240 min). Records of pregnant patients, individuals with craniofacial deformities, upper airway tumors, restrictive pulmonary pathology, a history of tuberculosis, incomplete clinical information, or evidence of prior OSAHS treatment with continuous positive airway pressure were excluded. The data used in this research are not publicly available, however, they can be provided by the authors upon request. The main characteristics of the dataset are shown in Table 2. The study protocol was approved by the Ethic committee of Libre University. The absence of informed consent was approved since this study solely included retrospectively gathered clinical variables of anonymous patients. Out of the 601 patients in the dataset, 511 received a positive diagnosis, while 90 were classified as healthy. To address this imbalance, we employed the SMOTE technique, which involves oversampling by generating new instances that closely resemble those in the dataset. This is in contrast to random oversampling, where data are duplicated until an equal number of instances are reached for each class. Following the balancing process, the dataset expanded to 1022 instances, evenly distributed with 511 instances for each class.

### 2.2. Models for the Diagnosis of OSAHS

#### 2.2.1. Model Proposed Utilizing Neural Networks

**Figure 2:**
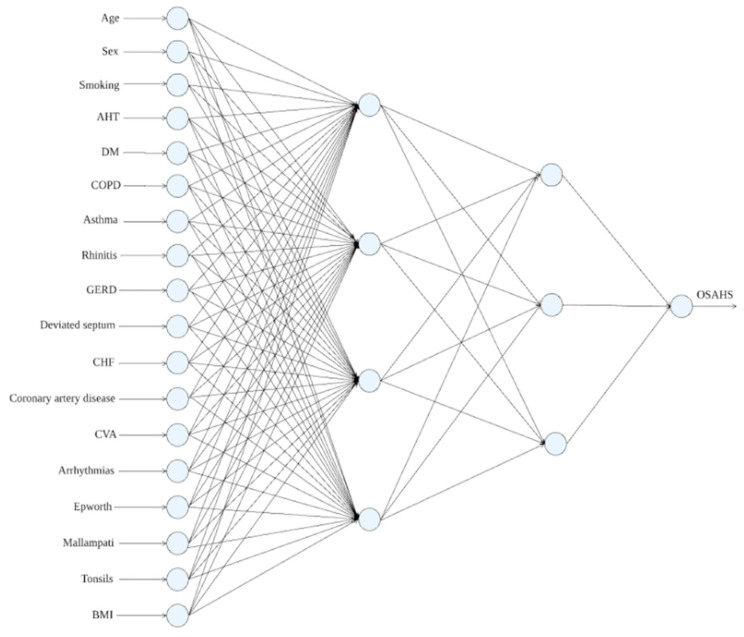
Proposed model utilizing a neural network.

Neural networks draw inspiration from the human nervous system, modeled with neurons and synaptic weights. These networks excel at learning through a training process using datasets where each instance is represented by independent variables and a class attribute. Adjusting synaptic weights during training enables artificial neural networks to establish connections between inputs and outputs, enabling predictions for class attributes in test instances. This study employed the MLP Classifier class from scikit-learn, utilizing the GridSearchCV function for hyperparameter tuning. Exhaustive tests covered activation functions (identity, logistic, tanh, and ReLU), solvers (Adam, lbfgs, and SGD), alpha values (0 to 1 in increments of 0.1), and three hidden layers (1 to 20 neurons each). Figure 2 illustrates a neural network designed for OSAHS diagnosis, featuring an 18-4-3-1 topology. This architecture encompasses 18 input layer neurons representing patient variables, followed by a first hidden layer with four neurons, a second hidden layer with three neurons, and a single neuron in the output layer indicating OSAHS presence.

#### 2.2.2. Model Proposed Utilizing Decision Trees

Decision trees, a machine learning technique, feature internal nodes and leaves. Internal nodes conduct tests on independent variables describing a patient, while leaves provide classifications indicating health or OSAHS diagnosis. To predict obstructive sleep apnea syndrome, the tree is traversed from top to leaf, classifying the patient at that node. The DecisionTreeClassifier class from scikit-learn, along with the GridSearchCV function, was used for hyperparameter optimization during experiments. Hyperparameter variations in the decision tree model included class_weight (‘balanced’ or ‘None’), criterion (‘entropy’ or ‘gini’), max_features (‘auto’, ‘log2’, or ‘None’), and max_depth (tested from 10 to 200 with increments of 10). Class_weight addresses class imbalances, criterion determines impurity calculation during node splitting, and max_features controls the number of features considered before node splitting. These variations enhance the model’s performance in predicting OSAHS in new patients.

#### 2.2.3. Proposed Model Utilizing Random Forests

The random forest model’s training process involves generating N trees, each evaluating 18 patient-specific values for OSAHS prediction. The model produces N diagnoses, and a majority criterion determines the final decision. If most of the N trees indicate OSAHS, the patient is classified as OSAHS positive; if the majority suggests OSAHS negativity, that is the model output. This approach enhances accuracy compared to a single Decision Tree. The scikit-learn library’s RandomForestClassifier class, along with GridSearchCV, was employed in experiments. Hyperparameters like criterion (‘gini’ or ‘entropy’), n_estimators (number of trees in the ensemble, tested from 10 to 200 with increments of 10), and min_samples_leaf (minimum samples for node split, explored from 1 to 5) were fine-tuned for optimal performance.

#### 2.2.4. Proposed Model Utilizing Extra Trees

In this study, we investigated the Extra Trees algorithm as an alternative ensemble technique. In contrast to Random Forests, Extra Trees constructs N trees from the entire training set, with each tree featuring randomly selected attributes in internal nodes. This inherent randomness facilitates diverse tree classifications within the same dataset, contributing to enhanced ensemble performance. The experimentation phase utilized the ExtraTreesClassifier class from the scikit-learn library. For tuning the hyperparameters criterion, n_estimators, and min_samples_leaf, the same options as those used for Random Forests were employed.

## 3. Results

### 3.1. Evaluation of Models for OSAHS Diagnosis

Table 3 presents the results obtained through the application of the four techniques on the balanced dataset. We employed several evaluation metrics, including accuracy ((TP + TN)/(TP + TN + FP + FN)), sensitivity (TP/(TP + FN)), specificity (TN/(TN + FP)), and the area under the ROC curve (AUROC). In this study, a True Positive (TP) is recorded when the model correctly predicts a patient with OSAHS, while a True Negative (TN) corresponds to an accurate prediction of a healthy individual. A False Positive (FP) is registered when the model incorrectly predicts OSAHS in a patient who is actually healthy. Finally, a False Negative (FN) occurs when the model incorrectly identifies a person as healthy when that person actually has OSAHS. These metrics were calculated on the test dataset, comprising 106 instances for both OSAHS negative and OSAHS positive.

In terms of specificity, the Random Forest and Extra Trees techniques achieve values of 84.0% and 89.6%, respectively. Conversely, the models generated with Neural Networks and Decision Trees exhibit lower specificities of 45.3% and 52.8%, respectively, indicating a challenge in accurately identifying healthy patients. However, all four models demonstrate higher sensitivity than specificity, suggesting a notable ability to correctly detect patients with OSAHS. Notably, the Random Forest model achieves the highest sensitivity at 94.3% among the four techniques. The area under the ROC curve reflects a balanced performance in predicting both classes, particularly evident in the Random Forest and Extra Trees techniques, which both achieve similar values of 89.2% and 89.6%, respectively.

Table 4 outlines the hyperparameters utilized during experimentation for each machine learning technique, along with the optimal hyperparameters obtained through GridSearchCV. We recommend employing these hyperparameter combinations to achieve the values presented in Table 3. However, given potential variations across datasets, we also advise conducting a fine-tuned hyperparameter adjustment process when working with different datasets.

### 3.2. Web Application for OSAHS Diagnosis

**Figure 3:**
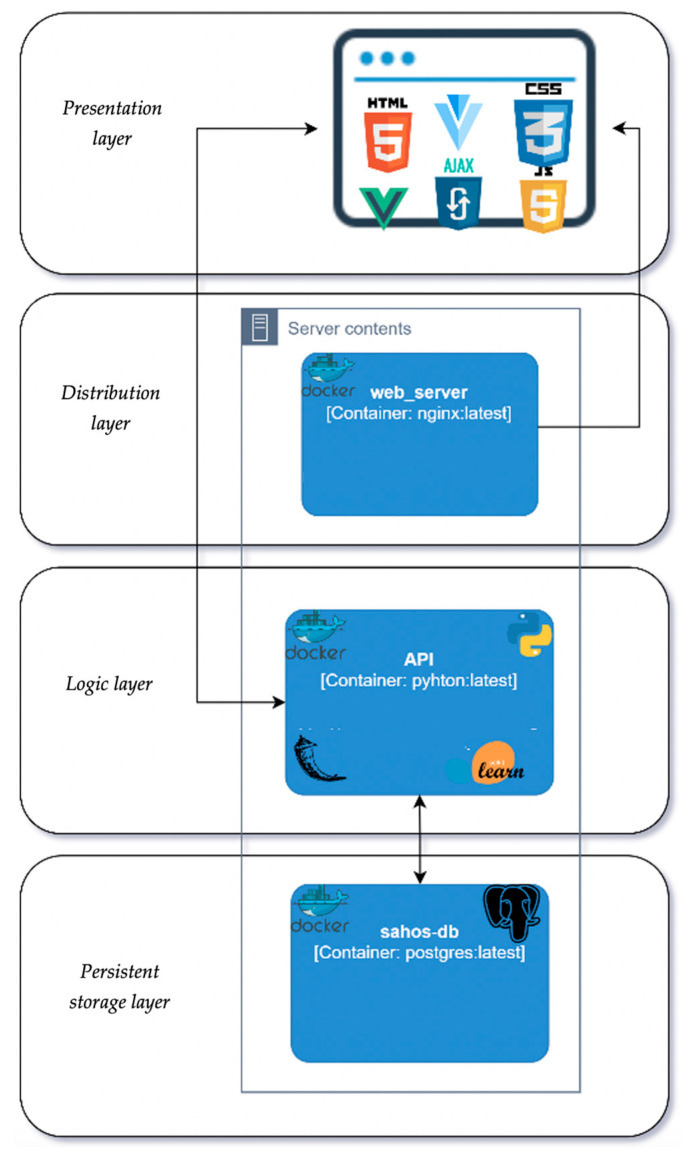
Software architecture for the OSAHS diagnosis.

A web application was created to deploy the proposed optimal model, ensuring accessibility for qualified medical professionals. Employing the Model-View-Controller architecture, an extra layer was added to integrate the OSAHS diagnosis model. Figure 3 illustrates the application architecture, comprising four layers: persistent storage, logical, distribution, and presentation. The chosen Random Forests model is seamlessly incorporated into the architecture. Designed to be responsive across various devices, the application utilizes the VueJS framework (version 2.6.11) and JavaScript for the presentation layer. Python, along with the Flask framework (version 2.0.1), handles logic, and Jupyter Notebook aids in script development. The scikit-learn library (version 0.24.2) and Python Joblib library (version 1.2.0) are crucial for model training and seamless integration into the application. The software not only aids in OSAHS diagnosis but also supports patient management by allowing input, deletion, or modification of patient data. It keeps a record of predictions from the machine learning model, and qualified medical professionals can affirm or reject the AI diagnosis post PSG, enabling potential model retraining.

**Figure 4:**
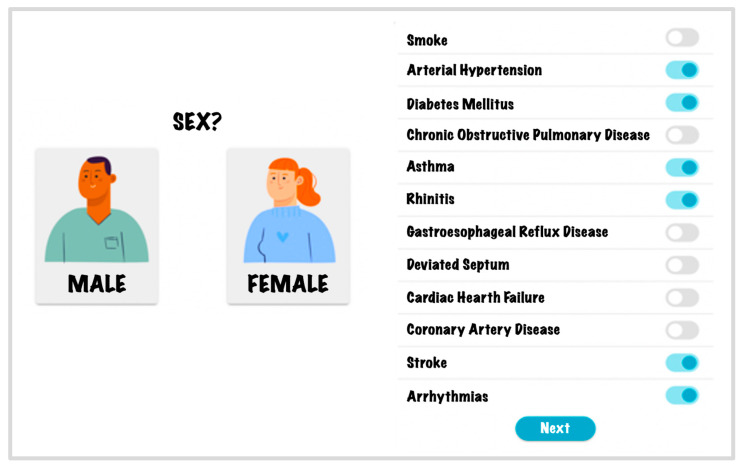
Data entry for OSAHS diagnosis.

Figure 4 illustrates a portion of the patient data input interface within the web application. Specifically, boolean variables like ‘Smoke’ and ‘Asthma’ are inputted into the application using on/off or switch buttons. However, other variables such as ‘BMI’ and ‘Mallampati’, which involve real numbers, are entered through a text box. Finally, variables like ‘Epworth’ and ‘Tonsils’, which consist of integer values, are entered via a slider with selectable values. After inputting the variables, the OSAHS diagnosis is conducted using the Random Forest model, and the prediction is displayed within the application interface. The application is not publicly available because a validation process is pending. However, the source code will be made available by the authors upon request.

## 4. Discussion

Based on the findings, the Random Forest technique demonstrates superior performance in screening for OSAHS, with an area under the ROC curve of 89.2% and a sensitivity of 94.3%, the highest among all metrics obtained in our experimentation. Consequently, we recommend employing Random Forest for OSAHS diagnosis. Overall, our experimentation highlights that ensemble methods, such as Random Forests and Extra Trees, consistently outperform other techniques across all metrics utilized in this study, showcasing significant differences in some cases. For instance, the area under the ROC curve for Random Forest and Extra Trees techniques achieve values of 89.2% and 89.6%, respectively, while Neural Networks and Decision Trees only reach 66.5% and 77.1%, respectively.

The obtained values in this research align with those documented in the existing literature concerning algorithms based on clinical variables. In some instances, it even surpasses them, emphasizing the effectiveness of this approach. For instance, in [10], the Neural Network technique achieves an accuracy of 86.6%, whereas in our investigation, it achieves 66.5%, likely due to variations in variables and datasets. However, our study introduces additional techniques that surpass even this high accuracy. Furthermore, models utilizing the Naive Bayes technique in [11] achieve an accuracy of 67.68%, a figure notably exceeded by the models proposed in our work.

In [22], the support vector machines technique (SVM) attained an AUROC of 0.82 and a sensitivity of 74.14% using data from 7830 patients at the National Taiwan University Hospital. While the current study yielded a lower AUROC with Neural Networks and Decision Trees, higher values were observed when employing ensemble methods, specifically Random Forests and Extra Trees, compared to the results reported in [22]. The work presented in [23] was based on using SVM, reaching a sensitivity of 0.80 and a recall of 0.93. These values are surpassed in the present study using the Random Forest technique. This variability emphasizes the necessity of interpreting comparative results with careful consideration of the specific context and dataset characteristics. However, the findings of this study suggest that ensemble methods and a balanced dataset contribute to enhanced results, aligning with the observations presented in [24].

We acknowledge some limitations in our study. Firstly, our reliance on medical record data may have impacted data quality. In fact, certain variables known to predict OSAHS, such as neck circumference [25], were omitted due to incomplete records. Secondly, our database was derived from a population with a high clinical suspicion of OSAHS, leading to a prevalence of positive cases in PSG. Consequently, our models may demonstrate higher sensitivity than specificity. Nonetheless, our study aimed to develop a screening tool for high-risk OSAHS patients, and thus, the dataset reflects this population. Lastly, the machine learning techniques utilized may not generalize effectively to new populations. potentially exhibiting overfitting to the original data. Therefore, external validation is crucial.

Regarding the developed web application, it enables efficient OSAHS screening, facilitating the prioritization of patients in need of PSG. This approach, utilizing machine learning for OSAHS diagnosis, has the potential to prioritize patients who would benefit most from the screening process, thereby reducing the rate of PSG negativity for OSAHS and enhancing accessibility and cost-effectiveness within the healthcare system. However, it is important to note that the software is currently tailored for use within the Colombian population, as the Random Forests model was trained exclusively on data from this demographic. As for the software itself, our plans include undergoing a validation process and gathering additional data for model retraining, with the aim of enhancing prediction accuracy.

## 5. Conclusions

The accuracy values of the four machine learning methods employed in this research for diagnosing OSAHS were 66.5% with Neural Networks, 71.7% with Decision Trees, 89.2% with Random Forests, and 89.6% with Extra Trees. Additionally, other metrics such as sensitivity range from 87.7% with Neural Networks to 94.3% with Random Forests. These results provide evidence that diagnosing OSAHS in the Colombian context is achievable. Notably, this study represents the first of its kind in Colombia, employing artificial intelligence for OSAHS diagnosis. While various machine learning techniques have been reported in other countries, it was imperative to train models with data representative of the Colombian population.

The experimentation also enabled us to conclude that ensemble methods, such as Random Forests and Extra Trees, consistently outperform Neural Networks and Decision Trees across all metrics utilized in this study. The optimal hyperparameters identified in this research suggest that 180 estimators are suitable for Random Forests, while 20 estimators are optimal for Extra Trees. These configurations, along with other combinations of hyperparameters, yield an area under the ROC curve of 89.2% for Random Forests and 89.6% for Extra Trees.

Another significant achievement of this research is that machine learning models relying on clinical parameters offer a valuable means for diagnosing OSAHS and can be seamlessly integrated into medical practice via web or smartphone applications. In this study, the developed application based on Random Forest enables efficient OSAHS screening with a sensitivity of 94.3%, allowing for the prioritization of patients in need of PSG. This innovation holds promise for reducing the number of PSGs negative for OSAHS and consequently increasing their availability, enhancing early diagnosis, and decreasing costs for the healthcare system.

## Figures and Tables

**Table 1:** Patient representation attributes.

Index	Variable	Definition	Data Type
1	Age	Age of the patient	Positive Integer
2	Sex	Gender of the patient	1—Masculine 0—Feminine
3	Smoking	Smoking status	Boolean
4	AHT	Presence or absence of diagnosed arterial hypertension	Boolean
5	DM	Diabetes Mellitus diagnosis status	Boolean
6	COPD	Chronic Obstructive Pulmonary Disease status	Boolean
7	Asthma	Presence or absence of diagnosed asthma	Boolean
8	Rhinitis	Presence or absence of diagnosed rhinitis	Boolean
9	GERD	Presence or absence of diagnosed Gastroesophageal Reflux Disease	Boolean
10	Deviated septum	Presence or absence of nasal septum deviation diagnosis	Boolean
11	CHF	Presence or absence of diagnosed chronic heart failure	Boolean
12	Coronary artery disease	Presence or absence of diagnosed coronary artery disease	Boolean
13	CVA	Ever diagnosed or not with stroke	Boolean
14	Arrhythmias	Presence or absence of diagnosed arrhythmias	Boolean
15	Epworth	Epworth Sleepiness Scale Score	Positive Integer
16	Mallampati	Mallampati Score rating	Real number
17	Tonsils	Brodsky Classification	Positive Integer
18	BMI	Body Mass Index	Real Number
19	OSAHS	OSAHS diagnosis	0—OSAHS negative 1—OSAHS positive

The table footer is (AHT = Arterial Hypertension; DM = Diabetes Mellitus; COPD = Chronic Obstructive Pulmonary Disease; GERD = Diagnosed Gastroesophageal Reflux Disease; CHF = Chronic Heart Failure; CVA = Cerebral Vascular Accident; BMI = Body Mass Index).

**Table 2:** Demographic and clinical characteristics of the study population.

Characteristics	n = 601 (%)
Age—average in years, standard deviation	51.80 ± 13.73
Sex	
Male	299 (49.7%)
Female	302 (50.3%)
Comorbidities	
Smoke	7 (1.16%)
Arterial Hypertension	275 (45.9%)
Diabetes Mellitus	82 (13.6%)
Chronic Obstructive Pulmonary Disease	31 (5.15%)
Asthma	54 (9.0%)
Rhinitis	60 (10.1%)
Gastroesophageal Reflux Disease	57 (9.5%)
Deviated Septum	69 (11.5%)
Heart Failure	16 (2.6%)
Coronary Artery Disease	26 (4.6%)
Stroke	13 (2.3%)
Arrhythmias	29 (4.9%)
Epworth Scale	9.59 ± 4.71
Normal < 11 points	372 (61.8%)
Very probable Certainty < 16 points	149 (24.7%)
Excessive Somnolence ≥ 16 points	80 (13.5%)
Body Mass Index—Average in kg/m^2^	30.85 ± 5.78
Underweight	2 (0.3%)
Normal	68 (11.3%)
Overweight	236 (39.3%)
Obesity Grade WHO	295 (49.1%)
Grade I	148 (24.6%)
Grade II	98 (16.3%)
Grade III	49 (8.2%)
Mallampati	
Class I	1 (0.18%)
Class II	16 (2.66%)
Class III	145 (24.19%)
Class IV	439 (73.04%)
Tonsils Brodsky Classification	
Grade 0	30 (4.99%)
Grade I	469 (78.03%)
Grade II	71 (11.81%)
Grade III	30 (4.99%)
Grade IV	1 (0.18%)
Sleep Latency—min	24.37 ± 22.01
Sleep Effectiveness—average in %, standard deviation	84.70 ± 38.16
Total Sleep Time—average in minutes	352.39 ± 135.45
Microarousal rate—average hourly rate, standard deviation	27.58 ± 20.35
Hypopnea Index—average hourly rate, standard deviation	22.45 ± 22.00
Duration of respiratory events—average in seconds, standard deviation	22.25 ± 7.42
Average oxygen saturation during REM sleep—average in %, standard deviation	92.71 ± 5.36
Average oxygen saturation during NON-REM sleep—average in %, standard deviation	93.69 ± 2.63
Average oxygen saturation during monitoring—average in %, standard deviation	94.48 ± 2.61
Average oxygen saturation during respiratory events—average in %, standard deviation	89.66 ± 6.23
T90—average in %, standard deviation	7.42 ± 16.71
Sleep time with snoring—average in minutes, standard deviation	11.47 ± 12.53
Minimum oxygen saturation during sleep—average in %, standard deviation	81.11 ± 10.61
T90 during REM sleep—average in minutes, standard deviation	8.73 ± 44.1
T90 during Non-REM sleep—average in minutes, standard deviation	21.51 ± 55.47
Hypopnea Index in supine—average in hours, standard deviation	28.43 ± 26.17
IAH in right side—average in hours, standard deviation	11.93 ± 24.80
IAH in left side—average in hours, standard deviation	1.90 ± 2.47

**Table 3:** Results obtained through the application of the four techniques on the balanced dataset.

Machine Learning Technique	Accuracy (95% CI)	Sensitivity (95% CI)	Specificity (95% CI)	Positive Predictive Value (95% CI)	Negative Predictive Value (95% CI)	Positive Likelihood Ratio (95% CI)	Negative Likelihood Ratio (95% CI)	AUROC
Neural Networks	0.665 (59.7–72.8%)	0.877 (79.9–93.3%)	0.453 (35.6–55.2%)	0.616 (57.1–65.9%)	0.787 (68.0–86.5%)	1.60 (1.33–1.93)	0.27 (0.16–0.47)	0.665
Decision Trees	0.717 (65.1–77.7%)	0.906 (83.3–95.4%)	0.528 (42.9–62.6%)	0.657 (60.9–70.3%)	0.848 (75.1–91.2%)	1.92 (1.56–2.37)	0.18 (0.10–0.33)	0.717
Random Forests	0.892 (84.1–93.0%)	0.943 (88.1–97.9%)	0.840 (75.6–90.4%)	0.855 (79.1–90.1%)	0.937 (87.7–97.0%)	5.88 (3.80–9.12)	0.07 (0.03–0.15)	0.892
Extra Trees	0.896 (84.7–93.4%)	0.896 (82.2–94.7%)	0.896 (82.2–94.7%)	0.896 (83.1–93.8%)	0.896 (83.1–93.8%)	8.64 (4.92–15.17)	0.12 (0.07–0.20)	0.896

**Table 4:** Optimal hyperparameters found during experimentation.

ML Technique	Hyperparameters Used during Experimentation	Optimal Hyperparameters Found
Neural Networks	Activation functions: identity, logistic, tanh, ReLU	activation: ReLU
	Solvers: Adam, lbfgs, SGD	solver: lbfgs
	Alpha values: 0 to 1 with increments of 0.1	alpha: 0.1
	Number of hidden layers: 1 to 3	Number of hidden layers: 2
	Number of neurons by layer: 1 to 20	Hidden_layer_sizes: (6,2)
Decision Trees	Class_weight: balanced, None	Class_weight: balanced
	Criterion: entropy, gini	criterion: entropy
	Max_features: auto, log2, None	splitter: random
	max_depth: 10 to 200 with increments of 10	max_depth:120
Random Forests	Criterion: gini, entropy	Criterion: gini, entropy
	n_estimators: 10 to 200 with increments of 10	n_estimators: 180
	min_samples_leaf: 1 to 5	min_samples_leaf: 1
Extra Trees	Criterion: gini, entropy	criterion: gini
	n_estimators: 10 to 200 with increments of 10	n_estimators: 20
	min_samples_leaf: 1 to 5	min_samples_leaf: 1

## Data Availability

The raw data supporting the conclusions of this article will be made available by the authors on request.
